# Gene therapy for hereditary hematological disorders: From clinical breakthroughs to future horizons

**DOI:** 10.1016/j.omtn.2026.103075

**Published:** 2026-08-27

**Authors:** Zhenghe Chen, Wei Wang, Youlan Wu, Yawen Qiang, Weisheng Cheng, Fang Liu

**Affiliations:** 1 Department of Clinical Laboratory, the First Affiliated Hospital of Anhui Medical University, No. 218 Jixi Road, Hefei, Anhui 230022, China; 2 Prenatal Diagnosis Center, Department of Obstetrics and Gynecology, the First Affiliated Hospital of Anhui Medical University, No. 218 Jixi Road, Hefei, Anhui 230022, China; 3 Department of Pathology, the First Affiliated Hospital of Anhui Medical University, No. 218 Jixi Road, Hefei, Anhui 230022, China; 4 NHC Key Laboratory of Study on Abnormal Gametes and Reproductive Tract, Anhui Medical University, No. 81 Meishan Road, Hefei, Anhui 230032, China; 5 Engineering Research Center of Biopreservation and Artificial Organs, Ministry of Education, No. 81 Meishan Road, Hefei, Anhui 230032, China; 6 Anhui Province Key Laboratory of Zoonoses, Anhui Medical University, No. 81 Meishan Road, Hefei, Anhui 230032, China; 7 The Provincial Key Laboratory of Zoonoses of High Institutions in Anhui, Anhui Medical University, No. 81 Meishan Road, Hefei, Anhui 230032, China

**Keywords:** MT: Oligonucleotides: Therapies and Applications, gene therapy, hereditary hematological disorders, sickle cell disease, thalassemia, hemophilia

## Abstract

Hereditary hematological disorders, including sickle cell disease, β-thalassemia, and hemophilia, are severe monogenic diseases that impose substantial morbidity and lifelong treatment burdens. Conventional therapies are largely supportive and rarely curative, whereas gene therapy is increasingly transforming the therapeutic landscape by addressing the underlying genetic defects. This review summarizes the major gene therapy strategies currently being developed for hereditary hematological disorders, with a particular focus on gene addition, gene editing, and gene silencing, as well as *ex vivo* and *in vivo* delivery platforms. We highlight recent clinical breakthroughs, including approved products for hemoglobinopathies and hemophilia, and discuss emerging approaches such as base editing, prime editing, epigenetic modulation, and lipid nanoparticle-mediated delivery. In addition, we examine the major challenges that continue to limit broader clinical adoption, including immune responses, off-target effects, conditioning-related toxicity, manufacturing complexity, high cost, and limited accessibility. Finally, we outline future directions that may accelerate clinical translation, including improved editing precision, next-generation vector engineering, artificial intelligence-assisted design, scalable manufacturing, and more equitable access to treatment. Together, these advances suggest that gene therapy is steadily moving from experimental innovation toward durable, potentially curative treatment for hereditary hematological disorders.

## Graphical abstract

**Figure:**
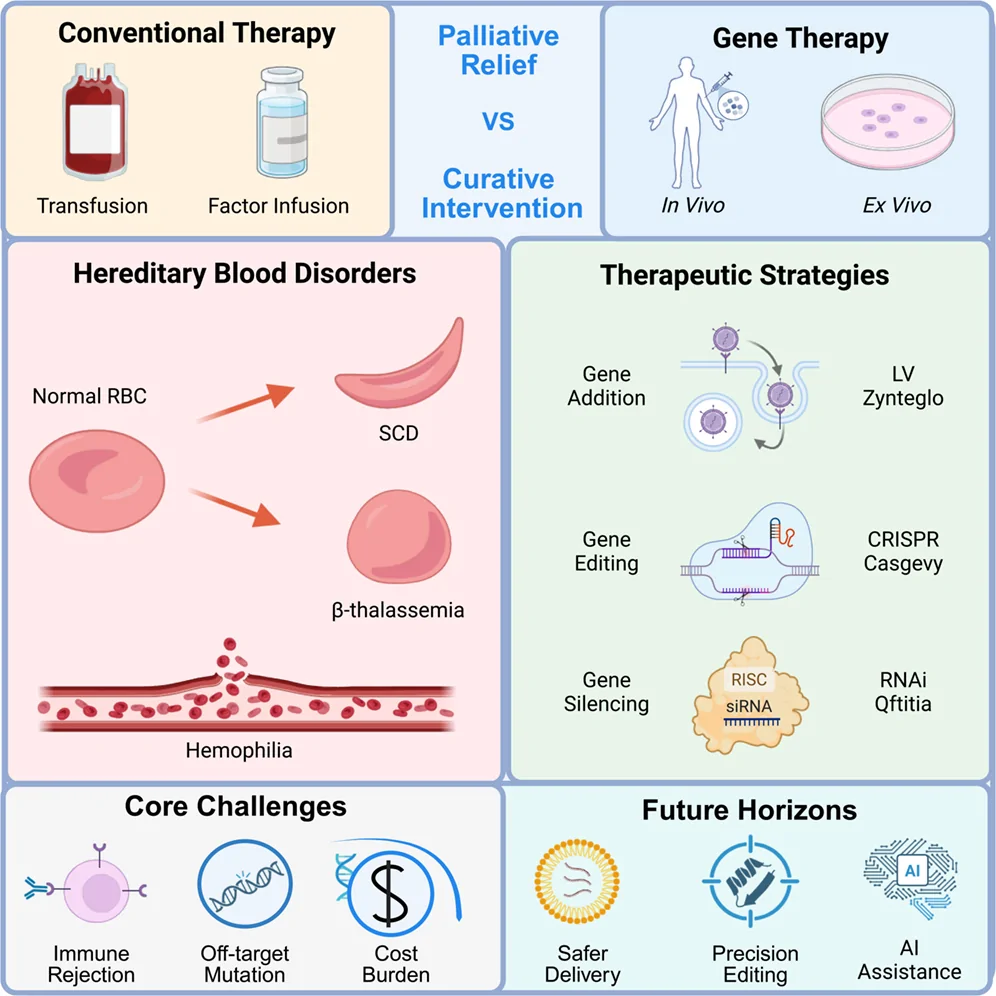

## Teaser

Gene therapy is transforming hereditary hematological disorders. This review summarizes approved gene addition, editing, and silencing strategies for sickle cell disease, thalassemia, and hemophilia, highlights curative potential, and discusses remaining challenges such as immune responses, cost, and accessibility.

## Introduction

Hereditary hematological disorders comprise a diverse group of monogenic diseases that impose substantial clinical and economic burdens worldwide. Driven by germline mutations, deletions, or chromosomal abnormalities, these lifelong conditions disrupt erythroid, myeloid, or megakaryocyte development, leading to chronic complications, organ damage, and reduced quality of life. Based on the affected hematopoietic lineage, these disorders are divided into three main categories. The first and most prevalent are hemoglobinopathies, including sickle cell disease (SCD) and β-thalassemia, caused by mutations in globin genes that disrupt normal hemoglobin production. A second group comprises immune-related blood disorders such as chronic granulomatous disease (CGD), which primarily affect phagocyte function and are beyond the scope of this review.^1^ The third major category is bleeding disorders, most commonly hemophilia A and B, resulting from loss-of-function mutations in coagulation factor genes.^2^ This review provides a comprehensive overview of recent progress, clinical translation and remaining challenges of gene therapy for the three most prevalent and clinically representative hereditary hematological disorders: SCD, β-thalassemia, and hemophilia.

Although traditional treatments, including replacement therapy and blood transfusion, relieve symptoms, they cannot achieve a radical cure. Long-term or lifelong management places heavy physical, mental, and financial burdens on patients. For some hereditary hematological diseases, allogeneic hematopoietic stem cell transplantation (allo-HSCT) offers a potential curative option, but graft-versus-host disease (GVHD) remains its main complication and a leading cause of mortality.^3^ GVHD triggers chronic inflammatory or fibrotic lesions, requiring long-term immunosuppression that raises infection and toxicity risks. High-intensity peri-transplant immunosuppression further exacerbates GVHD, making it a leading cause of post-transplant morbidity and mortality.^4^

Over the past three decades, gene therapy has advanced rapidly from early genetic supplementation to mature precision gene editing, with two landmark milestones: the first T lymphocyte-targeted clinical trial for adenosine deaminase deficiency (ADA-SCID) in 1990, and the FDA approval of Casgevy, the first CRISPR-Cas9 gene therapy in 2023.^5^^,^^6^

This review systematically summarizes the latest research advances in gene therapy for hereditary hematological disorders, with a focus on the technical principles, clinical translation progress, and core efficacy data of three mainstream therapeutic strategies (gene addition, gene editing, and gene silencing) in the treatment of hemoglobinopathies and hemophilia. Gene silencing approaches are discussed only briefly where clinically relevant, as their current clinical footprint in these disorders remains limited relative to gene addition and editing. Subsequently, we analyze the core technical, clinical, and accessibility challenges facing the clinical translation of these therapies, and finally outline the field’s future development directions, aiming to provide a systematic reference for the research and clinical application of gene therapy for hereditary hematological disorders.

## Technologies and strategies of gene therapy

### Classification of gene therapy

The inherent limitations of conventional supportive care have driven the development of gene therapy as a potentially curative alternative for hereditary hematological disorders, with three core therapeutic strategies now advanced to clinical translation: gene addition, gene editing, and gene silencing.

#### Gene addition technologies

The core principle of gene addition therapy is to deliver exogenous normal genes to compensate for the dysfunction of endogenous defective genes, without altering their sequences. For example, in the treatment of β-thalassemia, lentiviral vectors (LV) are used to introduce normal β-globin genes into HSCs. Optimized regulatory elements enable specific and efficient β-globin expression, correcting the anemic phenotype in mouse models.^7^ In ADA-SCID, retroviral vectors are applied to express the adenosine deaminase gene in HSCs. After transplantation, this approach effectively reconstitutes patients’ immune system and improves clinical outcomes. This strategy has become the most mature clinical translation modality for hereditary hematological disorders, with multiple products approved by the U.S. Food and Drug Administration (FDA) and National Medical Products Administration (NMPA) for hemoglobinopathies and hemophilia.^8^

#### Gene editing strategies

Gene editing enables precise correction of pathogenic mutations by directly modifying a patient’s defective endogenous genes, eliminating the need for exogenous gene supplementation. As the technology has evolved, editing tools have expanded from nuclease-induced double-strand breaks (DSBs) to include single-base modifications and template-free gene writing, greatly broadening the scope of gene therapy applications.^9^ Mechanistically, gene editing introduces site-specific DSBs at target genomic loci, which then activate endogenous repair pathways such as homology-directed repair (HDR) and non-homologous end joining (NHEJ).^10^ Error-prone NHEJ often causes random indels and gene knockout, while HDR mediates precise sequence modification by relying on an exogenous DNA template.^11^ Serving as the primary mechanism for site-specific correction of pathogenic point mutations, HDR has great potential for treating hereditary hematological diseases. Its technical characteristics and clinical applications, particularly in SCD, are elaborated in later sections.

Three generations of core gene editing technologies have been developed: zinc-finger nucleases (ZFNs), transcription activator-like effector nucleases (TALENs), and CRISPR-Cas9. ZFNs, the first to enter clinical use, consist of a zinc-finger DNA-binding domain and a FokI cleavage domain. They have been applied in *CCR5* knockout for HIV therapy and *BCL11A* editing for SCD. However, ZFNs face constraints related to sequence-dependent design and relatively high off-target toxicity, making them better suited for clinical contexts with established long-term safety data.^12^ TALENs improve upon DNA recognition using transcription activator-like effector (TALE) domains, offering enhanced specificity and editing efficiency, along with better compatibility in human pluripotent stem cells. They have shown value in research on polycystic kidney disease (PKD) and hemoglobinopathies, though the complexity of protein assembly limits their convenience, restricting their use to precision editing in sensitive cell types.^13^ CRISPR-Cas9, which relies on a single guide RNA (sgRNA) and Cas9 nuclease, has transformed the field with its simplicity, cost-effectiveness, and versatility. Despite limitations such as PAM sequence requirements and off-target effects, it remains the most widely adopted gene editing platform.

Despite their broad application, most of these technologies rely on DSB induction, which has driven the development of newer, more precise editing tools. Base editing (BE) enables precise single-nucleotide substitutions without creating DSBs, thereby reducing off-target effects and chromosomal abnormalities. Base editors typically fuse a catalytically deficient Cas9 (dCas9) or nickase Cas9 (nCas9) with a deaminase. For example, adenine base editors such as the evolved dABE8e convert A·T to G·C pairs with high efficiency. The catalytically inactive dCas9 variant serves purely as a DNA-binding platform, directing the deaminase without cleaving DNA.^14^ Importantly, when dCas9 is fused to transcriptional effectors (e.g., CRISPRa/i), it can modulate gene expression without altering the DNA sequence.^15^ Thus, the dCas9 platform enables both permanent DNA editing and reversible epigenetic regulation. The first BE system was reported in 2016, offering a lower-risk option for point mutation diseases. 111^16^^,^^17^ Although BE avoids DNA double-strand cleavage, it still causes unwanted DNA off-target mutations and widespread RNA-level miscoding. Bystander editing at neighboring bases also impairs accuracy and limits it’s *in vivo* clinical use.^18^

Prime editing (PE) further expands editing capabilities, allowing arbitrary base substitutions, insertions, and deletions without the need for an exogenous DNA template. PE uses a fusion protein comprising a nickase Cas9 (nCas9) and a reverse transcriptase.^19^ Guided by a prime editing guide RNA (pegRNA), the complex targets the desired locus, where nCas9 creates a single-strand nick. The reverse transcriptase then uses the pegRNA’s extension as a template to directly reverse-transcribe the intended genetic information into the genome, with final editing completed by endogenous repair machinery.^19^^,^^20^

#### Gene silencing approaches

In contrast to gene editing, which introduces permanent genomic alterations, gene silencing mediates reversible, dose-dependent suppression of target gene expression without modifying the underlying DNA sequence. Through rational sequence design, gene silencing agents can selectively target pathogenic alleles with minimal off-target effects on normal genes. The core mechanism involves nucleic acid molecules hybridizing to disease-related mRNA, either inducing its degradation or inhibiting translation to block production of aberrant proteins.^21^ Three main platforms for therapeutic gene silencing have emerged: RNA interference (RNAi), antisense oligonucleotides (ASOs), and CRISPR interference (CRISPRi). RNAi utilizes small interfering RNA (siRNA) or short hairpin RNA (shRNA) to direct the RNA-induced silencing complex (RISC) to cleave target mRNA, achieving high knockdown efficiency but requiring careful optimization to avoid off-target silencing.^22^ ASOs are chemically modified single-stranded oligonucleotides that bind complementary mRNA to activate RNase H-mediated degradation or sterically block translation; 2′-O-methoxyethyl (MOE) and locked nucleic acid (LNA) modifications substantially improve their *in vivo* stability and targeting specificity.^23^ CRISPRi employs a catalytically inactive dCas9 guided by gRNA to occupy promoter regions and repress transcription initiation, enabling long-term, reversible and highly specific gene repression.^24^ Notably, Qfitlia (fitusiran), an RNAi therapeutic that targets antithrombin, was approved by the FDA in 2025 for routine prophylaxis to reduce bleeding episodes in patients with hemophilia A or B, with or without inhibitors.^25^ Lentiviral vector-delivered shRNA targeting *BCL11A* has also shown robust fetal hemoglobin reactivation in preclinical studies of SCD and β-thalassemia.^9^

#### *In vivo* and *ex vivo* gene therapy

Based on the site of genetic modification, current gene therapy is mainly divided into two major technical modalities: *ex vivo* and *in vivo*.^26^ The two modalities differ significantly in core implementation pathways, applicable scenarios, advantages, and limitations, and serve as a key classification dimension for adapting to the treatment needs of different diseases.

*Ex vivo* gene therapy takes the patient’s autologous HSCs as the core target cells. Target gene addition, editing, or silencing is completed *in vitro* first, and the modified cells are infused back into the patient to reconstitute normal hematopoietic function after expansion and quality control. It is currently the mainstream clinical approach of gene therapy for hereditary hematological disorders. However, conventional myeloablative conditioning brings multiple adverse complications, consisting of systemic treatment-associated toxicity, elevated infection risk, gonadal damage causing infertility, and irreversible long-term hematopoietic injury.^27^ Novel strategies including reduced-intensity conditioning and non-genotoxic conditioning are under development to overcome these obstacles.^28^

**Figure 1:**
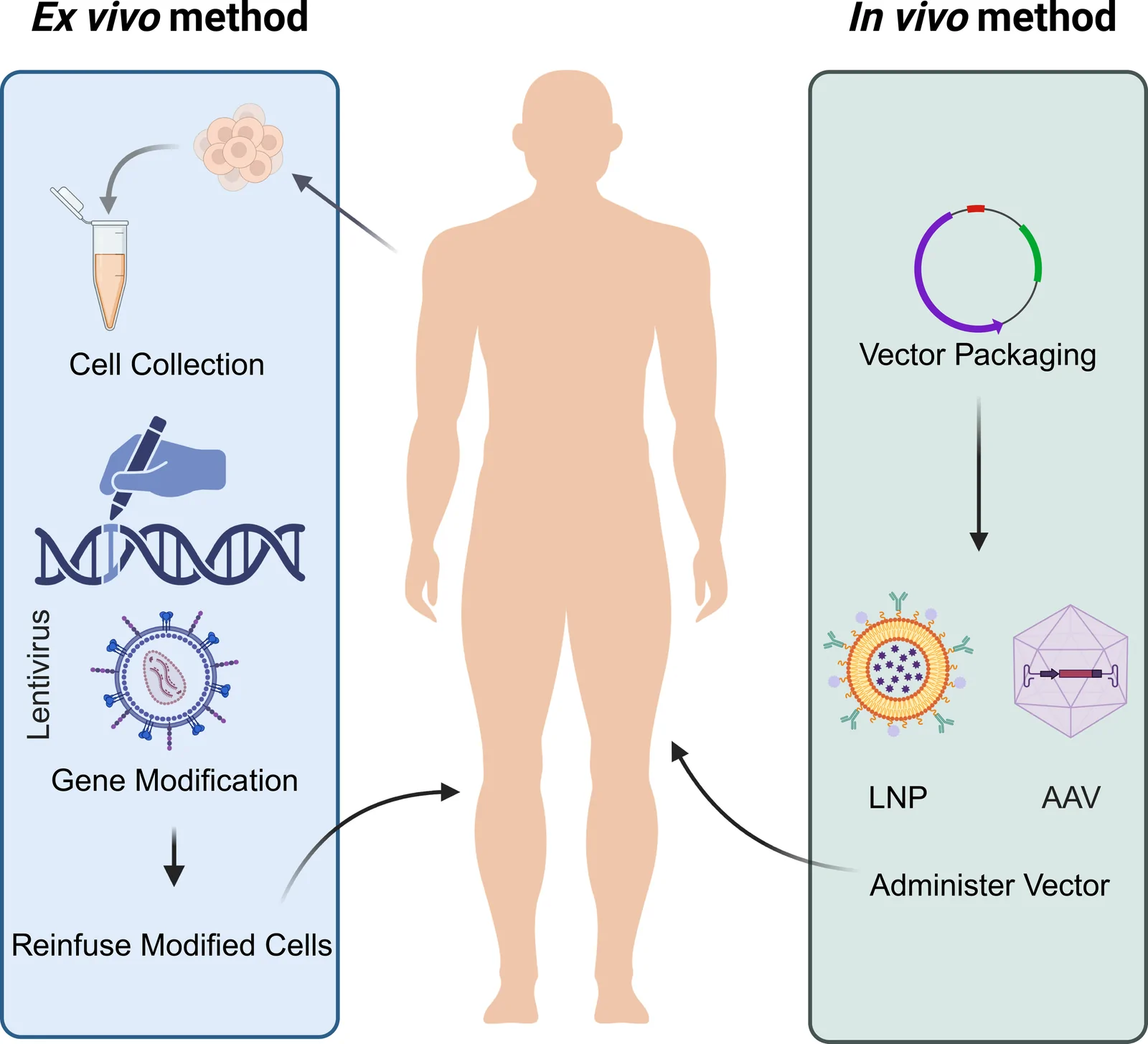
Schematic diagram of technical workflows for *ex vivo* and *in vivo* gene therapy Left image: *ex vivo* gene therapy: patients’ hematopoietic stem cells (HSCs) are harvested, genetically modified *ex vivo* using lentiviral vectors (LV) (gene addition, editing, or silencing), and reinfused after expansion and quality control. Right image: *in vivo* gene therapy: vectors (e.g., adeno-associated virus (AAV) or lipid nanoparticles (LNPs)) are directly administered into the patient to achieve *in situ* genetic modification.

In contrast, *in vivo* gene therapy directly targets the patient’s endogenous target cells to complete *in situ* genetic modification through delivery systems including engineered adeno-associated virus (AAV) vectors and LNPs. It eliminates the need for *ex vivo* cell manipulation and myeloablative conditioning, significantly reducing treatment-related toxicity and procedural complexity, and shows outstanding clinical application potential in populations intolerant to conventional conditioning (such as neonates).^29^^,^^30^ The core implementation workflows and differences between the two technical modalities are shown in Figure 1.

### Delivery modalities of gene therapy

The success of gene therapy is inherently dependent on vector systems capable of precisely delivering genetic material to target sites. Delivery systems represent both a foundational pillar and a major translational bottleneck for gene therapy, as they must protect nucleic acid cargo from degradation, traverse physiological barriers, and mediate either durable transgene expression or precise genome editing, forming the critical link between in *vitro* genetic constructs and *in vivo* therapeutic outcomes. From a clinical perspective, vector systems directly influence the efficacy and safety of gene therapy.^31^ From early retroviral vectors to the optimization and upgrading of LV and AAV vectors, and then to the gradual development of non-viral vectors, the direction of vector system iteration has shifted from merely achieving effective delivery to pursuing precise and safe delivery.

#### Viral vectors

Viruses have evolved to overcome multiple physiological barriers, making them ideal vectors for the delivery of genetic material in both *in vivo* and *ex vivo* gene therapy. In the gene therapy of hereditary hematological disorders, LV and AAV are the most widely used viral vectors.

LV is generated through multiple rounds of modification of lentiviruses such as HIV-1, retaining delivery functionality while eliminating pathogenicity. LV can accommodate exogenous genes of ≤10 kb, which meets the requirements of most gene therapy applications.^32^ Compared with γ-retroviral vectors, LV offers multiple advantages in *ex vivo* gene therapy for hematological disorders. Capable of transducing both dividing and non-dividing cells, LV features a safer integration profile with reduced tendency to target proto-oncogene promoters, which lowers the risk of insertional mutagenesis.^33^ The design of self-inactivating (SIN) LV, with deletion of viral enhancer and promoter elements within long terminal repeats (LTRs), further enhances biosafety by markedly reducing LTR-driven transactivation of adjacent genes.^34^ LV have been applied in numerous clinical trials for β-thalassemia and SCD, facilitating the marketing approval of cell products including Zynteglo (betibeglogene autotemcel, beti-cel) and Lyfgenia (lovotibeglogene autotemcel, lovo-cel). Notwithstanding these achievements, several challenges persist: clonal dominance caused by semi-random genomic integration, the mandatory use of myeloablative conditioning regimens, and the high costs associated with large-scale vector production.^35^^,^^36^

Native AAV is a non-enveloped single-stranded DNA virus, characterized by an icosahedral capsid with a diameter of approximately 25 nm and a genome size of ∼4.7 kb. The genome encodes replication-associated proteins (Rep) and capsid proteins (Cap), flanked by inverted terminal repeats (ITRs)-a key element mediating viral replication, packaging, and integration.^37^ Native AAV was first identified in 1960 as a contaminant in simian adenovirus preparations and cannot initiate its replication cycle independently, requiring coinfection with helper viruses (e.g., adenoviruses) for proliferation. To develop AAV as a gene delivery vehicle, researchers have engineered it into recombinant AAV (rAAV).^38^^,^^39^ The core design principle involves deleting the virus’s endogenous Rep and Cap-encoding genes while retaining only the ITRs as the vector backbone. This modification fundamentally abrogates the autonomous replication capacity of rAAVs and significantly reduces the risk of inducing host immune responses.^40^

In addition to the genome, the capsid plays a pivotal role in rAAV-mediated delivery, including encapsulating and protecting the genome, determining tissue tropism to enable precise targeting, and performing other critical functions. To improve the performance of AAV capsids, directed evolution and artificial intelligence (AI)-assisted engineering have achieved notable progress in recent years. In 2016, Viviana Gradinaru’s team developed an rAAV targeted evolution platform capable of generating rAAV capsids that specifically target Cre-expressing cell populations.^41^ Subsequently, in 2025, the same team launched the APPRAISE platform, which integrates AI to predict and design the relationship between capsid sequences and functional properties. This provides a strategy for engineering capsid proteins with optimal tropism and low immunogenicity.^42^ The core characteristics, therapeutic merits, limitations, and current clinical application status of commonly used AAV serotypes (such as AAV5 and AAV8) are comprehensively summarized in Table 1, which lays a fundamental basis for rational serotype selection in subsequent clinical translational practices of gene therapy.

**Table 1:** Characteristics of commonly used AAV serotypes in gene therapy for hereditary hematological disorders

Serotype	Target tissues	Main advantages	Main disadvantages	Clinical application status	Reference
AAV8	hepatocytes, skeletal muscle, heart	high hepatocyte transduction, Nab^+^ (20%–40%), high titer	no blood-brain barrier (BBB) penetration, cardiac less than AAV9	phase 1/2 for hemophilia B (not approved), preclinical for liver-based hematologic diseases	George et al.^43^, Reisset al.^44^
AAV9	systemic, central nervous system (CNS), heart, muscle	BBB penetration, high titer, abundant CNS data	weak hepatocyte vs AAV8, no significant HSPC tropism	approved for SMA (CNS), no phase 2 for hemoglobinopathies; only preclinical for β-thalassemia/SCD	Kang et al.^45^
AAVrh10	liver, CNS, heart	low Nab^+^ (15%–30%), non-human origin	moderate titer, limited safety data	phase 1/2 for CNS diseases, not a major vector for hemoglobinopathies	Pipe et al.^46^, Gernoux et al.^47^
AAV3A/3B	AAV3A, HSPC; AAV3B, hepatocytes	natural HSPC tropism (AAV3A), high hepatocyte (AAV3B)	low titer, manufacturing difficult	preclinical for *ex vivo* HSPC geneediting, not a clinical core vector	Brown et al.^48^, Chowdary et al.^49^
AAV6	HSPC, lung, heart, liver	high HSPC transduction (>90%), HDR donor delivery, lower Nab^+^ (15%–30%)	moderate immunogenicity, limited hepatic tropism	phase 3 hemophilia A (NCT03061201), preclinical HSPC editing	Wang et al.^37^, Zhong et al.^50^
AAV2	HSPC (*ex vivo*), CNS	best characterized, stable *ex vivo* transduction	Nab^+^ (60%–80%), not for systemic use	preclinical and research standard for *ex vivo* studies	Reiss et al.^44^
AAV5	hepatocytes, cardiomyocytes, neurons	lowest Nab^+^ (10%–20%), FDA/EU approved for hemophilia A & B	lower production titer than AAV8/9	approved (Hemgenix, Roctavian), not a vector for β-thalassemia/SCD	Pipe et al.^51^, Zhong et al.^52^

#### Non-viral vectors

Among non-viral vectors, LNPs stand out due to their unique advantages. LNPs not only shield nucleic acids from nuclease degradation but also enable efficient intracellular delivery via their pH-responsive properties.^35^ A pivotal milestone for LNP technology came in 2020, when LNP-based mRNA vaccines demonstrated remarkable efficacy during the COVID-19 pandemic: two leading formulations (BNT162b2 and mRNA-1273) achieved over 90% effectiveness, marking a transformative breakthrough in the translation of LNP platforms from preclinical research to clinical practice.^53^ The utility of LNPs in gene therapy is expanding rapidly, with substantial advancements, particularly in the treatment of hepatic disorders. The liver constitutes a natural target for LNP delivery, attributed to its dense vascular network and specialized endothelial architecture that promote LNP internalization. LNPs can encapsulate and deliver siRNA, mRNA, DNA, or gene-editing complexes, exerting therapeutic effects in liver diseases through mechanisms such as pathogenic gene silencing, therapeutic protein expression, or genetic defect correction.^54^ In the context of hematological disorders, a 2025 collaborative study between YolTech Therapeutics and East China Normal University focused on antibody-free targeted lipid nanoparticles (LNP-168). Via intravenous administration, the research team accomplished BE of the γ-globin gene (*HBG1/2*) promoter in murine HSCs. This intervention led to a 60% γ-globin activation rate in erythroid cells, effectively ameliorating the pathological phenotype in the diseased mouse model.^55^ In addition to delivering mRNA and gene-editing complexes, LNPs have been successfully used to encapsulate and deliver siRNA for liver-targeted gene silencing. For example, LNP-formulated siRNA targeting TTR (patisiran) has been approved for the treatment of hereditary transthyretin amyloidosis, reducing hepatic production of the pathogenic protein and demonstrating the clinical translatability of LNP-based RNAi therapeutics.^56^

## Research advances in gene therapy for hereditary hematological disorders

### Hemoglobinopathies

Hemoglobinopathies are a group of hereditary hematological disorders caused by abnormal hemoglobin structure or synthesis rate, often leading to chronic hemolytic anemia, organ damage, and severe impairment of patients’ health.^57^ The two most common subtypes are SCD and β-thalassemia, both caused by mutations or deletions in the globin gene cluster.^58^

#### Pathogenesis and conventional treatment limitations

**Figure 2:**
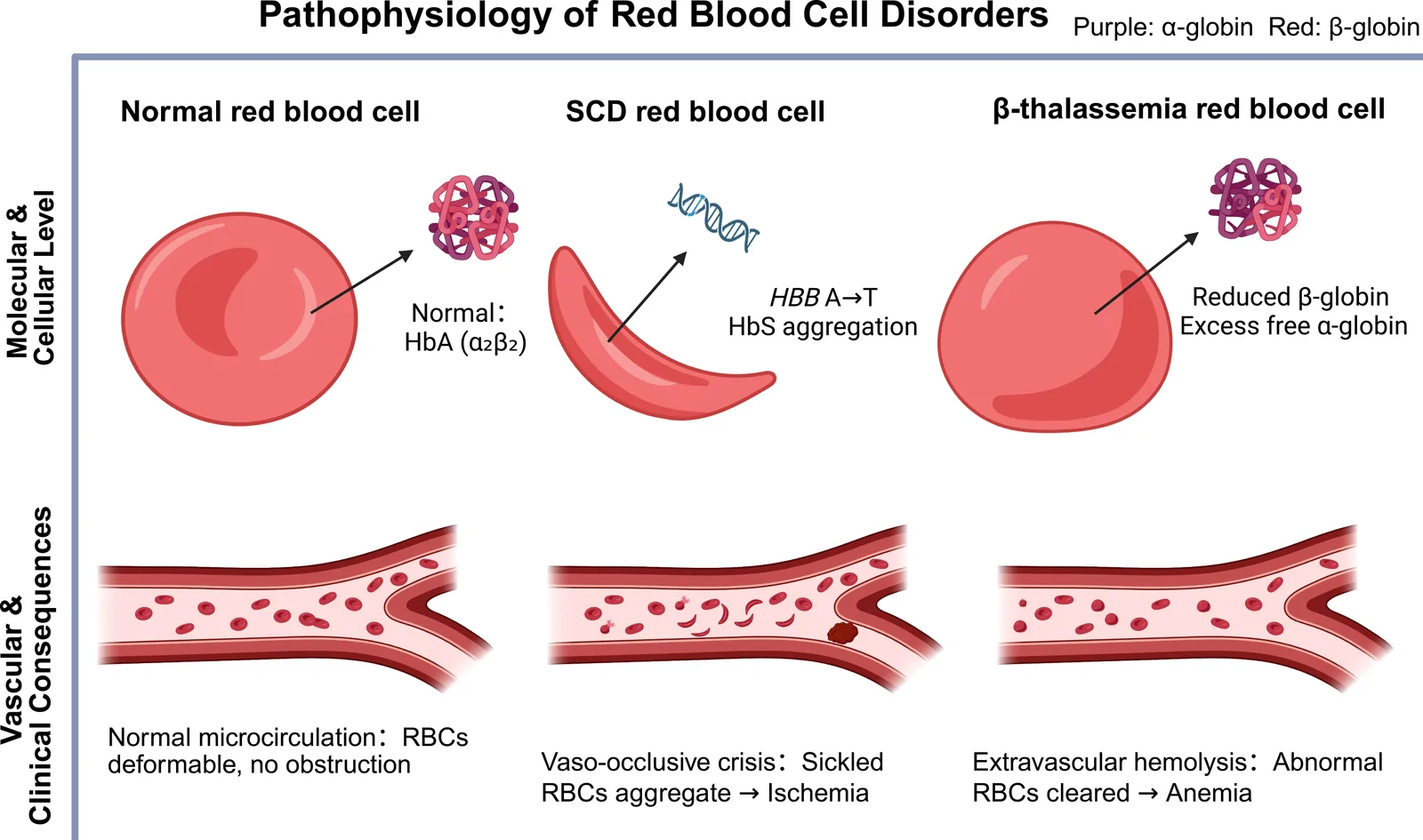
Pathophysiological comparison of normal, SCD, and β-thalassemia erythrocytes Left image: normal red blood cell (RBC) with normal hemoglobin and physiological microcirculation. Middle image: SCD RBC showing sickled morphology induced by HbS polymerization and subsequent vaso-occlusion. Right image: β-thalassemia RBC showing reduced β-globin chains, abnormal α-chain aggregation, erythrocyte malformation, and extravascular hemolysis.

Clinically, SCD is an autosomal recessive disorder resulting from a point mutation in the hemoglobin subunit beta (*HBB*) gene (GAG→GTG), leading to the synthesis of abnormal hemoglobin S (HbS) and erythrocyte sickling under hypoxic conditions.^57^ β-thalassemia is an autosomal recessive disorder caused by point mutations or small deletions in the *HBB* gene, resulting in reduced or absent β-globin chain synthesis and abnormal α/β-globin imbalance.^59^^,^^60^ The pathophysiological differences between normal erythrocytes, SCD erythrocytes, and β-thalassemia erythrocytes are visualized in Figure 2. Current conventional treatments for hemoglobinopathies are unable to achieve a radical cure, and are limited by transfusion-dependent iron overload, high risk of GVHD from allogeneic HSCT, and scarce HLA-matched donors, highlighting the urgent clinical need for curative gene therapy.^61^^,^^62^

#### Core clinical translational therapies

As the most mature technical route for hemoglobinopathy gene therapy, core clinical translational therapies include approved products and late-stage investigational candidates, covering two key curative directions: the fetal hemoglobin (HbF) activation strategy and the *HBB* gene addition strategy, both of which have achieved significant clinical benefits in clinical practice.

##### Approved core therapies

The most clinically advanced HbF reactivation strategy is exemplified by Casgevy (exagamglogene autotemcel, formerly CTX001), the first CRISPR-Cas9 gene therapy approved by the FDA for hemoglobinopathies. This non-viral *ex vivo* approach employs CRISPR-Cas9 to disrupt the erythroid-specific enhancer of *BCL11A* in autologous CD34^+^ hematopoietic stem and progenitor cells (HSPCs), thereby reactivating fetal hemoglobin production to compensate for the functional defect of adult hemoglobin. In the pivotal phase 3 CLIMB SCD-121 trial, 29 of 30 evaluable patients with SCD achieved freedom from severe vaso-occlusive crises (VOC) for at least 12 consecutive months, with all 30 patients free from VOC-related inpatient hospitalizations over the same period; HbF levels were stably maintained above 40% after treatment, with a mean of 43.9% ± 8.6% at month 6. Among 35 patients with transfusion-dependent β-thalassemia (TDT) in the parallel CLIMB THAL-111 trial, 32 achieved 12 months transfusion independence, with the long-term efficacy and safety of the therapy fully verified in multi-year follow-up.^63^^,^^64^^,^^65^ For hemoglobinopathies, *ex vivo* gene therapy has become a clinically mature approach that complements *in vivo* gene therapy strategies. In addition to strategies such as Casgevy that reactivate HbF via gene editing, the other main *ex vivo* approach uses viral vectors to deliver a functional globin gene into autologous HSPC (gene addition). Completed and ongoing clinical trials are summarized in Table 2.

**Table 2:** Clinical trials for gene addition in hemoglobinopathies

NCT Number	Other study ID numbers	Status	Enrollment	Interventions	Sponsor	Year
NCT03964792	P170006J	phase 1/2|active, not recruiting	6	DREPAGLOBE drug product	Assistance Publique - Hôpitaux de Paris	2019/11/12
NCT02151526	HGB-205	phase 1/2|completed	7	LentiGlobin BB305 (Zynteglo for β-thalassemia, Lyfgenia for SCD; both approved)	bluebird bio	2013/6/7
NCT01745120	HGB-204	phase 1/2|completed	19			2013/8/1
NCT02140554	HGB-206	phase 1/2|completed	54			2015/2/2
NCT02906202	HGB-207	phase 3|completed	24			2016/8/8
NCT03207009	HGB-212	phase 3|completed	19			2017/6/8
NCT04293185	HGB-210	phase 3|active, not recruiting	35			2020/2/14
NCT02186418	ARU-1801	phase 1/2|terminated	7	gamma-globin LV transduced autologous CD34^+^ HSCs	Children’s Hospital Medical Center, Cincinnati	2014/7/1
NCT06364774	22–020309	phase 1/2|recruiting	17	biological: ALS20	Children’s Hospital of Philadelphia	2025/4/14
NCT02247843	βAS3-FB	phase 1/2|active, not recruiting	4	βAS3-FB vector transduced CD34^+^ cells	Donald B. Kohn, M.D.	2014/12/1
NCT06399107	ESBI202493	phase 1/2|recruiting	85	gene therapy product after busulfan conditioning	Essen Biotech	2024/8/1
NCT05762510	2021–1101-001	early phase 1|recruiting	5	GMCN-508B (LentiRed) lentiviral genetic product	First Affiliated Hospital of Guangxi Medical University	2023/3/9
NCT06280378	CP-KL003-003/01	phase 1/2|recruiting	41	KL003 gene-modified cell injection product	Kanglin Biotechnology	2024/2/28
NCT06308159	LT02-101	phase 1/2|recruiting	6	Vebeglogene autotemcel	Lantu Biopharma	2024/5/11
NCT01639690	10–164	phase 1|active, not recruiting	10	TNS9.3.55 lentivirus transduced autologous CD34^+^ cells	San Rocco Therapeutics	2012/7/20
NCT06465550	BD-TDT-211005	phase 1|recruiting	9	BD211 genetic drug for HSC modification	Shanghai BDgene	2021/7/20

The *HBB* gene addition strategy is exemplified by two FDA-approved LV-mediated autologous HSPC gene therapies: Lyfgenia and Zynteglo. Lyfgenia delivers a modified βᴬ-T87Q globin gene into patients’ autologous HSPC, which can specifically inhibit the polymerization of HbS after expression in erythroid cells. Clinical data showed that 88% of evaluable patients with SCD achieved complete resolution of vaso-occlusive episodes 6–18 months after treatment, with no severe treatment-related adverse events such as insertional mutagenesis reported.^66^ Zynteglo, another classic product of the *HBB* gene addition strategy, uses the same βᴬ-T87Q globin lentiviral platform to restore functional β-globin synthesis. In the pivotal phase 3 HGB-207 trial for non-β^0^/β^0^ genotype TDT, 91% of evaluable patients achieved sustained transfusion independence for at least 12 months; notably, in the HGB-212 trial enrolling patients with the most severe β^0^/β^0^ genotype, the transfusion independence rate reached 92%, fully demonstrating the broad applicability and strong curative potential of this strategy.^67^^,^^68^

##### Investigational core therapies

Building on the clinical validation of approved HbF activation and *HBB* gene addition strategies, late-stage investigational candidates have further optimized editor design and therapeutic regimens, showing enhanced efficacy and safety in early clinical explorations. The HbF activation strategy also includes promising investigational candidates such as CS-101 injection, developed by Correct Sequence Biotech in collaboration with the First Affiliated Hospital of Guangxi Medical University. Based on the innovative transformed base editor (tBE) technology, CS-101 targets and edits the *BCL11A*-binding sites in the promoter regions of *HBG1/2* in patients’autologous HSPC, specifically relieving the transcriptional repression of *HBG* mediated by *BCL11A* and reactivating γ-globin expression.^69^ In a phase 1 trial (NCT06024876) of five patients with transfusion-dependent β-thalassemia, a single CS-101 infusion achieved median neutrophil and platelet engraftment at 16 and 25 days, respectively; all patients attained transfusion independence with a median time to last transfusion of 18 days. With a median follow-up of 23.0 months, total hemoglobin and HbF reached 12.4 ± 1.0 g/dL and 11.5 ± 0.9 g/dL at month 3, with F-cells exceeding 94.7%; editing efficiency remained stable at ∼34%–40%, with no detectable off-target edits across 145 sites.^70^ Adverse events were consistent with busulfan conditioning and autologous transplantation, with no deaths or cancer reported. These findings demonstrate that CS-101 can achieve rapid and durable transfusion independence.

The *HBB* gene addition strategy also has late-stage investigational candidates such as the GbGM gene vector, which encodes a modified *HBG* gene (HbF^G16D^) with enhanced anti-sickling activity. Combined with low-intensity melphalan conditioning, this strategy achieved more than 80% reduction in vaso-occlusive episodes in SCD patients in early clinical studies, and the expression level of modified HbF remained stable for a long time.^71^

##### Domestic investigational candidates

Parallel to global advances, domestic investigational candidates for hemoglobinopathies have also made breakthroughs, tailoring core technical logic to the genetic background and clinical needs of Chinese patients with SCD and β-thalassemia. HGI-001, a domestically developed lentiviral gene addition therapy, adopts the *HBB* gene addition strategy and uses the optimized Lenti*HBB*^T87Q^ vector to enhance erythroid-specific expression of functional β-globin.^72^ Following the same core technical logic as Zynteglo, it has demonstrated definitive efficacy in preclinical and early clinical studies for severe β-thalassemia (including β^0^/β^0^ genotype), effectively reducing patients’ transfusion dependence and providing a high-quality domestic alternative to imported products.^73^

#### Gene editing frontier therapies

Gene editing enables fundamental correction of pathogenic *HBB* mutations, representing the next-generation curative strategy for hemoglobinopathies, with relevant clinical trials summarized in Table 3. In the field of gene editing, BE offers a direct approach for precisely correcting pathogenic mutations. For example, the ABE8e-NRCH editor can accurately convert the disease-causing *HBB*^S^ allele into the non-pathogenic *HBB*^*G*^ (Makassar β-globin) variant. This method has achieved approximately 80% *ex vivo* editing efficiency in patient-derived CD34^+^ HSPC and sustained a conversion frequency of about 68% for 16 weeks in humanized mouse models, effectively restoring functional β-globin expression.^74^

**Table 3:** Clinical trials for gene editing in hemoglobinopathies

NCT Number	Other study ID numbers	Status	Enrollment	Interventions	Sponsor	Year
NCT03728322	*HBB* HSC-01	early phase 1|unknown status	12	iHSCs treatment group	Allife Medical Science and Technology	2019/1/1
NCT05456880	BTX-AUT-001	phase 1/2|recruiting	15	BEAM-101	Beam Therapeutics	2022/8/30
NCT04211480	2019-BRL-00CH1	not applicable|completed	6	γ-globin reactivated autologous HSCs	Bioray Laboratories	2020/10/1
NCT06300723	2024-BRL-101-SCD-01	not applicable|enrolling by invitation	3	BRL-101		2024/7/29
NCT06024876	CS-101–06	early phase 1|completed	5	CS-101	CorrectSequence Therapeutics	2023/8/26
NCT06291961	CS-101–01	phase 1|completed	9			2024/4/18
NCT07489196	CS-101–09	phase 2|not yet recruiting	20			2026/4/5
NCT06565026	CS-206–01	early phase 1|recruiting	5	CS-206		2024/9/2
NCT06647979	BCH-201	phase 1|not yet recruiting	10	*BCL11A* enhancer Cas9-edited CD34^+^ HSPCs	Daniel Bauer	2025/12/1
NCT04853576	EM-SCD-301–001	phase 1/2|active, not recruiting	45	EDIT-301	Editas Medicine	2021/4/21
NCT05444894	EM-301-BThal-001	phase 1/2|active, not recruiting	9			2022/4/29
NCT04819841	KMAU-001–001	phase 1/2|recruiting	15	nula-cel	Kamau Therapeutics	2021/3/29
NCT04774536	21–33287	phase 1/3|recruiting	9	CRISPR_SCD001	Mark Walters, MD	2024/9/18
NCT04443907	OTQ923	phase 1|terminated	4	OTQ923	Novartis Pharmaceuticals	2020/8/25
NCT03432364	ST-400–01	phase 1/2|completed	5	ST-400	Sangamo Therapeutics	2018/2/14
NCT03653247	003SCD101	phase 1/2|active, not recruiting	8	Plerixafor + Busulfan + BIVV003		2019/3/6
NCT05864170	HGI-001-CTP	early phase 1|recruiting	3	HGI-001	Shenzhen Hemogen	2022/5/27
NCT06655662	HGI-001C04	phase 1|recruiting	8			2024/6/12
NCT06506461	SAGES1	phase 1|recruiting	25	Plerixafor + Busulfan + Gene-modified CD34^+^ cells	St. Jude Children’s Research Hospital	2025/3/21
NCT03655678	CTX001-111	phase 2/3|completed	59	Casgevy (CTX001, approved)	Vertex Pharmaceuticals	2018/9/14
NCT03745287	CTX001-121	phase 2/3|completed	63			2018/11/27
NCT05329649	VX21-CTX001-151	phase 3|recruiting	15			2022/5/2
NCT05356195	VX21-CTX001-141	phase 3|recruiting	15			2022/5/3
NCT05477563	VX21-CTX001-161	phase 3|recruiting	26			2022/8/2

HDR-mediated gene correction serves as a viable alternative for precise genome manipulation. Distinct from error-prone NHEJ that generates random insertions and deletions, HDR relies on an exogenous DNA template to accomplish accurate sequence replacement or targeted insertion at the desired genomic locus.^75^ HDR preferentially functions in dividing cells and exhibits limited activity in quiescent HSCs; nevertheless, multiple innovative approaches, including engineered donor templates, small-molecule potentiators, and cell-cycle synchronization strategies, have greatly boosted HDR efficiency in therapeutically relevant cell populations.^76^^,^^77^ For SCD, HDR has been successfully applied to revert the disease-causing *HBB*^*S*^ allele to wild-type *HBB* in patient-derived HSPCs. The phase 1 CEDAR trial (NCT04819841), the first clinical investigation of HDR-based gene therapy for SCD, adopts CRISPR-Cas9 combined with an AAV6-delivered donor template to correct HbS to HbA in autologous CD34^+^ HSPCs.^78^ The first enrolled patient experienced a serious adverse event of prolonged pancytopenia. Encouragingly, 12 months long-term follow-up confirmed full recovery from the cytopenias, sustained gene correction, and no clonal dominance. Collectively, these findings demonstrate the promising prospects as well as remaining hurdles of HDR-based gene editing for inherited hematological diseases.^79^

Despite PE for hemoglobinopathies being in early clinical stages, proof-of-concept validation of its safety and efficacy in clinical trials for conditions like CGD provides a robust translational framework for *HBB* gene editing.^80^ Additionally, CRISPR-mediated epigenetic editing reactivates HbF expression by targeting CpG demethylation in the *HBG* promoter or regulating factors like UHRF1/MBD2. This strategy allows for controllable and reversible induction of HbF without altering the DNA sequence, offering a precise and low-toxicity therapeutic option.^81^^,^^82^

#### Gene silencing strategies for hemoglobinopathies

Notably, gene silencing strategies such as lentiviral-mediated shRNA targeting *BCL11A* have also shown promise in clinical exploration. By specifically inhibiting the expression of *BCL11A*, a key repressor of γ-globin, these strategies achieve HbF activation without altering genomic DNA sequences, thereby complementing gene editing and gene addition approaches.^9^ Clinical trial data in Table 4 demonstrate that such gene silencing regimens can reduce symptoms in SCD patients and improve transfusion independence in β-thalassemia, which highlights their value as part of combined therapeutic strategies.

**Table 4:** Clinical trials of gene silencing for hemoglobinopathies

NCT Number	Other study ID numbers	Status	Enrollment	Interventions	Sponsor	Year
NCT05353647	P00038082	phase 2|active, not recruiting	25	autologous CD34^+^ HSC cells transduced with the LV containing a shRNA targeting *BCL11A*	David Williams	2022/7/12
NCT03282656	P00026188	phase 1|active, not recruiting	10	single infusion of autologous bone marrow-derived CD34^+^ HSC cells transduced with the LV containing a short hairpin RNA targeting *BCL11A*		2025/12/1
NCT04091737	CSL200	phase 1|terminated	1	autologous enriched CD34^+^ cell fraction that contains CD34^+^ cells transduced with LV encoding human γ-globinG16D and short hairpin RNA 734	CSL Behring	2019/10/2

#### Novel delivery and combined strategies

##### *In vivo* LNP-mediated base editing

In the field of gene therapy for hemoglobinopathies, novel delivery systems and combined therapeutic strategies represent key directions for improving efficacy and accessibility, further advancing the clinical application of approaches such as HbF reactivation and *HBB* gene addition.

In terms of *in vivo* delivery for gene editing, antibody-free targeted LNPs represented by LNP-168 can achieve BE of the *HBG1/2* promoter in murine HSCs via intravenous injection. This delivery system offers a new pathway for HbF reactivation strategies, inducing approximately 60% γ-globin expression in erythroid cells and effectively ameliorating the pathological phenotypes of sickling and hemolysis in mouse disease models.^55^

##### Combined therapeutic strategies for hemoglobinopathies

Furthermore, combining gene editing with HbF reactivation may lead to superior therapeutic outcomes. This approach addresses key limitations of single strategies, such as insufficient HbF expression or incomplete mutation repair, by enabling simultaneous direct *HBB* gene correction and HbF reactivation. Preclinical studies have shown that this combined strategy maintains HbF expression stably above 30% in animal models and achieves complete transfusion independence, offering a new direction for the treatment of severe hemoglobinopathies.^74^^,^^83^

### Hemophilia

Similar to hemoglobinopathies, hemophilia is a monogenic hereditary hematological disorder caused by pathogenic mutations in coagulation factor genes. However, due to the structural differences between globin genes and coagulation factor genes, as well as distinct clinical phenotypes (hemolytic anemia vs. bleeding diathesis), the gene therapy strategies for hemophilia exhibit unique characteristics, with *in vivo* AAV-mediated gene addition as the most mature technical route. Hemophilia is an X-linked recessive disorder caused by mutations in the *F8* (coagulation factor Ⅷ) and *F9* (coagulation factor Ⅸ) genes on the X chromosome, leading to the deficiency of corresponding coagulation factors. According to the type of deficient coagulation factor, *F8* mutation-induced FⅧ deficiency causes hemophilia A, while *F9* mutation-induced FⅨ deficiency results in hemophilia B.^84^ Coagulation factor deficiency leads to delayed wound or postoperative healing, as well as recurrent spontaneous bleeding in joints and muscles, which severely impairs patients’ quality of life and long-term survival.

#### Pathogenesis and conventional treatment limitations

Clinically, hemophilia is classified by the activity level of coagulation factors: severe hemophilia is defined as coagulation factor activity < 1%, which manifests as recurrent spontaneous joint or muscle bleeding. The global incidence of hemophilia A is (5–10)/100,000 population, while the prevalence in China is (2.73–3.09)/100,000, with regional variations mainly related to diagnostic capacity and medical resource availability.

The *F8* gene is one of the largest genes in the human genome, with a full length of approximately 186,000 base pairs and encoding a protein of 2351 amino acids. The protein exhibits a domain structure of A1–A2–B–ap–A3–C1–C2, among which the B-domain is not essential for procoagulant function but contributes to efficient cellular secretion.^85^^,^^86^ In contrast, the *F9* gene has a significantly smaller coding region (∼1.4 kb), with a more compact structure that is more compatible with common viral vector delivery systems.^87^^,^^88^ Based on the structural differences between the FⅧ and FⅨ genes, differentiated vector design and technical routes have been formed for current gene therapies for hemophilia A and B, respectively.

Conventional treatment for hemophilia relies on lifelong regular infusion of exogenous coagulation factors, which can only control bleeding symptoms but cannot achieve a radical cure. Long-term infusion not only brings a heavy economic burden to patients but also carries a high risk of coagulation factor inhibitor formation, which is the most serious complication of replacement therapy.^89^ In rare cases, liver transplantation has been documented to correct hemophilia, because the liver is the primary source of factor VIII and factor IX. However, this approach carries high surgical risks, requires lifelong immunosuppression, and is only considered for patients with concomitant end-stage liver disease, making it unsuitable as a standard curative option.^90^^,^^91^ These limitations highlight the urgent need for a safe and durable curative strategy. Gene therapy has emerged as such an approach, offering the potential for a one-time functional cure by enabling sustained endogenous production of the missing coagulation factor.

#### AAV-mediated *in vivo* gene addition therapy

AAV-mediated *in vivo* gene addition is the most mature and widely used technical route for hemophilia gene therapy and is also the core platform for all currently approved hemophilia gene therapy products worldwide. Completed and ongoing *in vivo* clinical trials for hemophilia are detailed in Table 5, covering key candidates targeting both hemophilia A and B. This strategy delivers a functional *F8* or *F9* gene to hepatocytes via engineered AAV vectors, enabling sustained, stable expression of functional coagulation factors *in vivo* to restore the systemic coagulation cascade and achieve a one-time functional cure.

**Table 5:** *In vivo* clinical trials for hemophilia

NCT Number	Other Study ID Numbers	Status	Enrollment	Interventions	Sponsor	Study Start
NCT04676048	ASC-HA-001	phase 1/2|recruiting	12	AAV-mediated FVIII gene therapy	ASC Therapeutics	2022/8/3
NCT02576795	270–201	phase 1/2|completed	15	Roctavian (valoctocogene roxaparvovec, approved, Hemophilia A)	BioMarin Pharmaceutical	2015/9/28
NCT03370913	270–301	phase 3|completed	144			2017/12/19
NCT03520712	270–203	phase 1/2|terminated	3			2018/4/24
NCT04323098	270–303	phase 3|completed	22			2020/12/08
NCT02396342	CT-AMT-060–01	phase 1/2|completed	10	Hemgenix (etranacogene dezaparvovec-drlb, approved, Hemophilia B)	CSL Behring	2015/6/10
NCT03489291	CSL222_2001	phase 2|completed	3			2018/7/24
NCT03569891	CSL222_3001	phase 3|completed	67			2018/06/27
NCT06008938	CSL222_4001	not applicable	500 (Estimated)			2023/06/15
NCT04135300	GT2019001	not applicable|completed	10	Xinjiuning (bbm-h901, approved, Hemophilia B)	Institute of Hematology & Blood Diseases Hospital, China	2019/10/16
NCT05203679	BBM001-CLN1001	phase 1/2|recruiting	32			2021/12/30
NCT05709288	IIT2022051	phase 1|recruiting	9			2023/03/23
NCT04728841	IIT2020030	not applicable	12	GS001		2021/3/4
NCT05152732	IIT2021043	early phase 1|recruiting	3 (estimated)	VGB-R04		2021/12/28
NCT05454774	IIT2022019	early phase 1|recruiting	8	BBM 002		2022/7/19
NCT02484092	SPK-9001–101	phase 2|completed	15	BEQVEZ (fidanacogene elaparvovec, approved, Hemophilia B)	Pfizer	2015/11/18
NCT03061201	C3731001	phase 2|completed	13			2017/6/21
NCT03307980	C0371003	phase 2|active, not recruiting	21			2017/6/22
NCT03861273	C0371002	phase 3|active, not recruiting	51			2019/7/29
NCT04370054	C3731003	phase 3|active, not recruiting	76	*In vivo* AAV2/6-mediated FVIII gene therapy		2020/8/18
NCT06379789	R131L1265-HEMB-2318	phase 1/2|recruiting	130	REGV131 LNP1265	Regeneron Pharmaceuticals	2024/9/11
NCT05441553	VGB-R04-101	phase 1/2|unknown status	26	VGB-R04	Shanghai Vitalgen BioPharma	2022/7/1
NCT01620801	AAV8-hFIX19-101	phase 1|terminated	4	AAV8-hFIX19	Spark Therapeutics	2012/10/1
NCT03734588	SPK-8016–101	phase 1/2|completed	4	SPK-8016		2019/1/30
NCT03003533	SPK-8011–101	phase 1/2|completed	25	SPK-8011		2017/1/26
NCT06297486	SPK-8011–302	phase 3|withdrawn	0			2024/3/13
NCT03001830	UCL /13/0076	phase 1/2|active, not recruiting	14	AAV2/8-HLP-FVIII-V3	University College, London	2017/6/14
NCT03369444	UCL/15/0552	phase 1/2|terminated	10	FLT180a		2017/12/5
NCT02554773	LTE14762	phase 1/2|completed	34	Fitusiran	Genzyme, a Sanofi Company	2015/09/18
NCT03417102	EFC14768	phase 3|completed	60			2018/2/14
NCT03549871	EFC15110	phase 3|completed	80	Fitusiran	Genzyme, a Sanofi Company	2018/7/25
NCT06145373	SFY17741	phase 4|recruiting	20			2024/3/1

##### Approved therapies for hemophilia B

Hemgenix (etranacogene dezaparvovec) is the first FDA-approved one-time gene therapy for adult patients with severe hemophilia B. It uses an AAV5 vector to deliver a high-activity coagulation FIX Padua variant (R338L), whose coagulation activity is 5–8-fold higher than that of the wild-type, enabling therapeutic efficacy even at low expression levels.^46^^,^^92^ The key characteristics of AAV5, including its lower seroprevalence of neutralizing antibodies, are summarized in Table 1. The final analysis of the phase 3 HOPE-B study showed that at 4 years post-treatment, FIX activity remained stable at 29.2% in 54 treated patients, with a 93% reduction in annualized bleeding rate (ABR), and 94% of patients discontinued prophylactic coagulation factor infusions. The most common adverse event was reversible transaminase elevation, which could be rapidly alleviated with glucocorticoid intervention, with no vector-related severe adverse events reported.^51^

Beqvez (fidanacogene elaparvovec-dzkt) is an FDA-approved one-time gene therapy for adult patients with moderate to severe hemophilia B. To achieve efficient hepatic transduction and sustained endogenous FIX expression with minimal vector exposure, it uses an AAVrh74 var capsid vector carrying a codon-optimized high-activity FIX R338L transgene, which is driven by a liver-specific human α_1_-antitrypsin promoter and enables effective packaging and targeted expression in hepatocytes at a low dose of 5×10^11^ vector genome copies per kilogram of body weight.^93^ The phase 3 BENEGENE-2 study confirmed that the mean FIX activity was maintained at 26.9% in 45 treated patients 15 months after treatment, with 82% of patients retaining FIX activity above 5% at 24 months, a 71% reduction in ABR, and over 90% of patients eliminated reliance on regular prophylactic FIX infusions. The safety profile was consistent with previous phase 1–2a studies, with the most common adverse event being reversible transaminase elevation, no vector-related severe liver injury or insertional mutagenesis reported, and no infusion-related serious adverse events, thrombotic events, development of FIX inhibitors, or malignant conditions observed.^94^

Hemgenix has broader applicability for hemophilia B, being approved for patients regardless of AAV5 neutralizing antibody status and thus accessible to more people with pre-existing immunity. Beqvez, by contrast, requires negative AAVrh74 neutralizing antibody test results for enrollment, limiting its eligible population to a certain extent.^51^^,^^94^

Xinjiuning (dalnacogene ponparvovec injection), the first hemophilia B gene therapy product approved in China, represents a landmark clinical translation achievement of the *in vivo* AAV-mediated gene addition strategy in the hemophilia field.^95^ Consistent with the design logic of Hemgenix, this therapy uses BBM-H901, a novel engineered hepatotropic rAAV vector, to deliver an optimized human coagulation *F9* gene to hepatocytes, enabling sustained, stable expression and secretion of functional FIX to restore systemic coagulation activity. The tolerability, safety, and efficacy of Xinjiuning were systematically evaluated in a 2019 exploratory study (NCT04135300) and a 2021-initiated phase 3 trial (NCT05203679).^96^^,^^97^ A single intravenous infusion of BBM-H901 (5 × 10^12^ vector genomes per kilogram [vg/kg]) yielded a mean FIX activity of 55.1 IU/dL at week 52, with an ABR reduced to 0.6 (markedly below the pre-specified superiority threshold of 5.0). Eighty percent of patients remained bleed-free, with annual FIX replacement infusions dropping from 58.2 to 2.9. The only common adverse event was grade 1–2 glucocorticoid-reversible transaminase elevation; no treatment-related severe adverse events, FIX inhibitors, or thrombotic complications occurred during follow-up.^97^

##### Approved therapies for hemophilia A

Roctavian (valoctocogene roxaparvovec) is the first FDA-approved one-time gene therapy for adult patients with severe hemophilia A. To overcome the packaging capacity limitation of AAV vectors, it uses a B-domain-deleted (BDD) *F8* transgene, which reduces the coding region length to 4.4 kb. However, due to the large size of the *F8* expression cassette, the resulting vector genomes approach or exceed the optimal packaging limit of AAV, a known challenge for first-generation hemophilia A gene therapies.^86^^,^^98^ The phase 3 GENEr8-1 study confirmed that FVIII activity was maintained at 11.3% in 134 treated patients 4 years after treatment, with a 93% reduction in ABR, and over 90% of patients eliminated reliance on regular prophylactic infusions.^99^ The safety profile was consistent with previous studies, with the most common adverse event being reversible transaminase elevation, no vector-related severe liver injury or insertional mutagenesis reported.

Although AAV8 exhibits significantly higher hepatic transduction efficiency than AAV5, Hemgenix and Roctavian both use AAV5 as the delivery vector. The core rationale for AAV5 selection includes its lower seroprevalence of pre-existing neutralizing antibodies (NAbs), favorable tolerability in hemophilia patients with comorbid fatty liver, milder immunogenicity, and reduced hepatotoxicity risk.^100^ For hereditary hematological disorder gene therapy, AAV serotypes with distinct tropism and manufacturing properties meet diverse clinical and research needs, with their key characteristics, advantages, limitations, and current clinical applications summarized in Table 1.

##### Investigational *in vivo* gene therapy candidates for hemophilia a in China

In parallel with global clinical translation progress, China has achieved breakthroughs in domestic hemophilia A gene therapy, represented by the investigational *in vivo* AAV8-based candidate GS001. In a completed phase 1 dose-escalation trial, 12 adult patients with severe hemophilia A received GS001 at 2×10^12^ vg/kg (low dose) or 4×10^12^ vg/kg (high dose), with prophylactic immunosuppression initiated 1 week before infusion. At week 104, the high-dose cohort achieved a median FVIII activity of 42.7 IU/dL, with all patients maintaining FVIII levels above 29 IU/dL; the median ABR dropped from 12.5 to 0, with a more than 95% reduction in FVIII replacement infusions. The regimen was well tolerated, with no FVIII inhibitor formation or treatment-related severe adverse events.^101^ The core innovations of GS001 include an optimized liver-specific expression cassette enabling high-efficiency FVIII expression at an ultra-low dose, and a prophylactic tacrolimus plus glucocorticoid regimen that effectively inhibits CD8^+^ T cell immune responses to maintain long-term transgene expression. Its multicenter phase 3 clinical trial is currently ongoing to further verify long-term safety and efficacy.

##### Analysis of approved gene therapy products for hereditary hematological disorders

For hemoglobinopathies that require HSC transplantation to reconstitute hematopoietic function, *ex vivo* therapeutic manipulation is currently the only clinically approved approach. Casgevy, a gene editing-based product, achieves therapeutic efficacy through targeted mutation correction, while Lyfgenia and Zynteglo, gene addition-based products, exert therapeutic effects via functional compensation of abnormal globin, forming a complementary portfolio of clinical options.

For hemophilia, coagulation factors produced by transduced hepatocytes can be secreted and exert systemic therapeutic effects. Accordingly, AAV-mediated *in vivo* gene addition has become the mainstream of approved products. The domestically developed product Xinjiuning carries an optimized human FIX transgene, which achieves higher post-treatment FIX activity compared with international products, and is offered at a substantially lower domestic price, resulting in improved clinical accessibility relative to imported products. A detailed comparison of the aforementioned approved products regarding technical route, indication, core therapeutic mechanism, key advantages, and critical limitations is provided in Table 6.

**Table 6:** Approved gene therapy products for hereditary hematologic disorders

Product Name	Approving agency	Core indications	Technical route	Key clinical efficacy data	Core safety data	Applicable population	Core technical shortcomings
Casgevy	FDA/EMA	SCD, TDT β-thalassemia	gene editing (CRISPR-Cas9-mediated *BCL11A* knockout) + non-viral vector + *ex vivo*	97% SCD patients free of severe VOC (12 m), HbF≥40%; 91% TDT patients with transfusion independence (12 m)	no severe TRAEs; occasional mild myelosuppression	SCD (all genotypes), TDT β-thalassemia (including β^0^/β^0^)	myeloablative conditioning required for *ex vivo* therapy, not suitable for elderly/infirm patients; extremely high cost
Lyfgenia	FDA	SCD	gene addition (modified βᴬ-T87Q globin) + LV + *ex vivo*	88% SCD patients free of VOC (6–18 m); stable HbF expression	black box warning for insertional mutagenesis; 2 AML cases reported; no GVHD	SCD (moderate-severe, recurrent VOC)	potential insertional mutagenesis risk of LV; *ex vivo* therapy dependent on HSPC collection
Zynteglo	FDA/EMA	TDT β-thalassemia	gene addition (modified βᴬ-T87Q globin) + LV + *ex vivo*	92% β^0^/β^0^ TDT with transfusion independence (12 m); 91% non-β^0^/β^0^ TDT with transfusion independence (12 m)	no severe TRAEs, occasional mild infection	TDT β-thalassemia (including all genotypes)	difficult mass production of LV; insufficient globin expression in some patients
Hemgenix	FDA	severe hemophilia B	gene addition (FIX Padua variant) + AAV5 + *in vivo*	FIX activity stably at 29.2% (4 y); 93% ABR reduction; 94% discontinued prophylactic infusion	reversible transaminase elevation, no vector-related severe TRAEs	adults with moderate-severe hemophilia B (FIX activity ≤2%); no restriction on AAV5 NAbs status	low transduction efficiency of AAV5; NAbs^+^ rate 10%–20%; not suitable for children
BEQVEZ	FDA	severe hemophilia B	gene addition (high-activity FIX-Padua variant) + AAVRh74var vector + *in vivo*	FIX activity ∼26.9% (15 m); 71% ABR reduction; >90% stopped prophylaxis	mild reversible transaminase elevation, no insertional mutagenesis or severe hepatic injury	adult males with moderate-severe hemophilia B (FIX activity ≤2%), negative for AAVRh74var NAbs, no FIX inhibitors	pre-existing AAVRh74var NAbs excluded (∼10%–20% prevalence); long-term follow-up ongoing
Roctavian	FDA/EMA	severe Hemophilia A	gene addition (BDD-*F8*) + AAV5 + *in vivo*	FVIII activity stably at 11.3% (4 y); 93% ABR reduction; 90% discontinued prophylactic infusion	reversible transaminase elevation, no insertional mutagenesis or liver injury	adult patients with severe hemophilia A (FVIII activity <1%)	AAV5 packaging capacity limitation, requiring B-domain deletion of *F8*; pre-existing NAbs exclude some patients
信玖凝(Xinjiuning)	NMPA	severe hemophilia B	gene addition (optimized human FIX) + self-developed AAV (BBM-H901) + *in vivo*	FIX activity 55.1 IU/dL (52 w); ABR reduced to 0.6; 80% patients bleed-free	grade 1–2 reversible transaminase elevation; no FIX inhibitors or thrombotic complications	adult/adolescent patients with severe hemophilia B (FIX activity <1%)	lack of long-term safety data (>5 years); mass production process of self-developed AAV to be optimized
Qfitlia	FDA	hemophilia A/B	gene silencing (RNAi) + LNP + *in vivo*	median ABR reduced to zero; >90% reduction vs. on-demand; >60% zero bleeding	mild transaminase elevation, injection-site reactions	adults/adolescents (≥12 years) with hemophilia A/B, with/without inhibitors	requires bi-monthly subcutaneous injection; non-curative, long-term prophylaxis

All currently approved products still have notable limitations: *ex vivo* therapies depend on pre-treatment myeloablative conditioning; *in vivo* AAV-based therapies are not eligible for patients with pre-existing AAV NAbs; and the high pricing of international products severely restricts widespread clinical accessibility. These limitations define the core directions for future product optimization.

#### RNAi-based non-factor prophylactic therapy

While gene silencing has seen more limited clinical application in hemoglobinopathies, it has achieved a major breakthrough in hemophilia with the approval of the first RNAi therapy. In addition to the one-time curative *in vivo* gene therapy mediated by AAV described previously, RNAi offers a novel, non-genome-editing, factor-independent long-term prophylactic approach for hemophilia. Fitusiran (Qfitlia) is the first approved RNAi therapy for hemophilia, administered subcutaneously once every two months. Key clinical results from phase 3 trials showed that the median ABR was reduced to zero, representing a >90% reduction compared with conventional on-demand therapy, and over 60% of patients achieved zero bleeding throughout the year.^102^ The safety profile is favorable, with only mild, transient elevations in liver enzymes and injection-site reactions as manageable adverse events. This approach fills the clinical gap for non-curative long-term prophylaxis without permanent genomic modification, although it requires repeated administration.^103^ The one-time curative strategy for hemophilia remains centered on AAV-mediated *in vivo* gene addition therapy.

#### *Ex vivo* gene therapy strategies

Complementing *in vivo* modalities, *ex vivo* gene therapy addresses the core limitation of pre-existing AAV antibodies, providing a viable option for patients excluded from current *in vivo* gene therapy. *Ex vivo* gene therapy for hemophilia predominantly uses LV to deliver functional *F8* or *F9* genes into the patient’s autologous HSPC, with related clinical trials detailed in Table S1. After *in vitro* genetic modification, expansion, and strict quality control, the modified cells are infused back into the patient after myeloablative conditioning to achieve stable engraftment in the bone marrow. This strategy circumvents the core limitation of pre-existing AAV NAbs *in vivo* AAV therapy, making it suitable for patients with high AAV antibody titers who are ineligible for AAV-mediated treatment. It is recognized as a critical complementary regimen to *in vivo* AAV therapy, as well as an exploratory platform for next-generation technologies such as HSPC-based gene editing.^104^ For future development, this modality can be combined with more precise gene editing technologies to achieve *in situ* correction of coagulation factor genes in autologous HSPC. This approach can realize more physiological expression regulation driven by endogenous promoters, which is superior to exogenous promoter-driven gene addition strategies; however, it requires a longer R&D cycle and more rigorous long-term safety verification.

#### *Ex vivo* gene editing strategies for hemophilia

Compared with gene addition strategy, gene editing can achieve permanent correction of pathogenic *F8*/*F9* mutations at the endogenous gene locus, avoiding the risk of random insertion and long-term expression instability of exogenous genes, and has become a key research direction in the field of hemophilia gene therapy. Currently, BE and PE have achieved breakthroughs in preclinical studies for hemophilia: base editors can precisely correct single-nucleotide pathogenic mutations in F9 without inducing DNA DSBs, achieving stable FIX expression in preclinical mouse and non-human primate models.^105^ PE enables precise correction of large fragment deletions and complex mutations in *F8*, expanding the scope of eligible patients for gene editing therapy.^106^

Another promising *ex vivo* gene editing approach is the use of CRISPR-Cas9 to engineer B lymphocytes into therapeutic protein-secreting cells. It uses CRISPR-Cas9 to insert the FIX-Padua transgene into the *CCR5* safe harbor locus in a patient’s autologous B cells. After these cells differentiate into plasma cells, they secrete highly active FIX. In animal studies, FIX was detectable one day after a single dose, reached steady state within two weeks, and lasted for over 184 days. Re-dosing was possible without preconditioning, and only very low-frequency off-target sites were found.^107^ Together, these *ex vivo* gene editing platforms offer a potential new therapy for hemophilia that is durable and allows repeat dosing.

#### Novel non-viral delivery strategies

Non-viral vector systems have garnered increasing attention in hemophilia gene therapy. The Rychak team developed a liver-targeted, non-viral *in vivo* gene-insertion approach using the piggyBac DNA transposon system. Compared with conventional AAV vectors, this platform enables delivery of large transgenes, achieves stable and efficient genomic integration of therapeutic transgenes, and has the potential to adjust FVIII activity through repeated administration.^108^ To further optimize delivery efficiency and safety, researchers developed novel co-encapsulated LNP formulations that co-deliver super piggyBac (SPB) transposase mRNA and transposon plasmid DNA.^109^ In juvenile wild-type mice, hFVIII antigen expression was 50% higher than that of the dual-nanoparticle approach. The incorporation of N-acetylgalactosamine (GalNAc)-targeting ligands reduced proinflammatory serum cytokine levels by 2–3 orders of magnitude, avoiding transaminase elevation while maintaining high hFVIII expression. These advancements lay a crucial foundation for the clinical translation of non-viral gene therapy.^109^^,^^110^

The approval of multiple AAV-mediated gene therapies has achieved a functional cure for hemophilia, but pre-existing AAV antibodies, vector-related immune responses, and the limited durability of FVIII/ FIX expression are still key bottlenecks restricting the clinical popularization of these therapies.

## Challenges and future directions of gene therapy

### Safety concerns of gene therapy

#### Immune responses

The primary safety concern in gene therapy is immune responses, which may induce severe adverse reactions or even be life-threatening. The sources of immune responses primarily involve the vector itself and the genetic material it carries. As the most commonly used vector in gene therapy, AAV exhibits lower immunogenicity than other vectors; however, severe immune responses may still occur with high-dose systemic administration.^111^ A key limitation of AAV-based therapies is the development of immune memory following initial exposure. Neutralizing antibodies induced by the first dose can persist for years, effectively preventing successful re-administration of the same AAV vector, which is a major barrier to long-term treatment strategies, especially if transgene expression declines over time.^112^ Immune responses against the transgenic product itself are less frequently reported, but they can also lead to serious clinical consequences. Foreign genetic material, acting as a foreign antigen, can trigger immune responses in the body. Additionally, the presence of pre-existing antibodies limits patients’ eligibility for AAV vector-based therapies.^113^ Pre-existing immunity, together with treatment-induced memory B and T cells, not only reduces primary efficacy but also precludes re-dosing, leaving no option for booster administration if therapeutic effect wanes.^114^ Currently, many clinical trials have used immunosuppressants to manage immune responses. While immunosuppressants can suppress immune reactions, they also increase the risk of systemic infections. Therefore, balancing immunosuppressant use or developing highly specific immunosuppressants has become a key research focus.

Besides humoral immunity, AAV elicits robust cellular immune responses as well. After *in vivo* delivery, AAV capsid-derived peptides are presented via major histocompatibility complex class I (MHC-I) on transduced cells, which subsequently activate CD8^+^ cytotoxic T lymphocytes (CTLs). These effector CTLs can recognize and clear hepatocytes or other target cells expressing AAV capsid proteins, resulting in diminished transgene expression and potential liver injury.^115^ Memory CD8^+^ T cells induced by the first AAV exposure can survive for a long time and undergo rapid clonal expansion upon re-encounter with AAV, severely impairing the safety and efficacy of re-dosing strategies.^116^ Even without repeated administration, persistent subclinical T-cell reactivity may slowly reduce the abundance of therapeutic proteins over months to years, weakening the long-term durability of gene therapy. Accordingly, suppression of both humoral and cellular immune memory is critical to maintain sustained therapeutic effects. Multiple innovative approaches, including rapamycin-mediated immune tolerance induction and rational design of AAV capsids with fewer T cell epitopes, are currently under intensive investigation.^114^^,^^117^

#### Insertional mutagenesis and carcinogenicity

Insertional mutagenesis represents a severe safety concern for integrative gene therapy vectors, with the potential to induce carcinogenicity. This issue emerged in early gene therapy clinical trials, spurring continuous advancements in vector technologies. The mechanism underlying insertional mutagenesis involves the semi-random integration of gene vectors into the host genome, which may activate proto-oncogenes or inactivate tumor suppressor genes. Studies have demonstrated that semi-random integration of transgenes into the chromosomal DNA of hematopoietic cells can trigger clonal competition and even induce leukemia or sarcoma. Adverse events associated with insertional mutagenesis have become a major barrier to the progressive development of gene therapy. Early gene therapy clinical trials fully exposed the severity of this risk. In trials of γ-retroviral vectors for the treatment of ADA-SCID, several patients developed post-treatment T cell leukemia. Subsequent investigations revealed that viral vectors integrated near the LMO2 proto-oncogene, leading to its aberrant activation and subsequent induction of leukemia.^118^

#### Vector targeting and off-target effects

Ideal gene therapy should precisely deliver therapeutic genes to target tissues or cells while avoiding expression in non-target tissues. Among the currently widely used viral vectors, AAV vectors exhibit relatively favorable tissue tropism, yet their natural tropism often fails to meet therapeutic needs. Different AAV serotypes exhibit distinct tissue preferences: for instance, AAV2 primarily targets the liver and eyes, while AAV9 demonstrates superior blood-brain barrier penetration capacity.^119^^,^^120^ Such natural tropism is often misaligned with therapeutic targets for specific diseases, leading to low-level expression of therapeutic genes in non-target tissues, which reduces therapeutic efficacy and poses concomitant safety risks.

Although the CRISPR-Cas9 system has greatly improved the precision of gene editing, its off-target effects remain a major safety challenge for clinical application. Off-target effects are unintended cleavage or modification at non-target sites by gene-editing tools, which may induce gene mutations, chromosomal abnormalities, or other adverse consequences.^121^ The off-target effects of CRISPR-Cas9 mainly stem from partial matching of the gRNA to multiple genomic loci. Studies have shown that Cas9 protein can bind to and cleave genomic sites with partial homology to the target sequence, and this binding relies on various non-canonical base-pairing interactions. Despite the availability of modern off-target detection methods, such as CIRCLE-seq, GUIDE-seq, and DISCOVER-seq, which can detect low-frequency off-target events, there is still room for improvement in their detection sensitivity and specificity.^122^

As next-generation gene-editing tools, base editors avoid generating DSBs; nevertheless, their off-target effects are equally non-negligible. Research data have shown that the classical cytosine base editor (CBE) induces an average of 1,353 single-nucleotide variants (SNVs) in transgenic mice, which is 5-fold higher than that in the control group.^123^ These off-target mutations occur not only at the DNA level but also extend to the RNA level: RNA sequencing results indicate that both BE3 and its high-fidelity optimized version YE1-BE3-FNLS induce transcriptome-wide RNA SNVs, and the number of RNA SNVs exhibits a positive correlation with the expression level of CBE.^123^^,^^124^ This indicates that even with optimized DNA-level editing precision, unintended RNA-level modifications may still emerge as new safety concerns, further increasing the complexity of technical risks.

### Patient individual variability and ethical-social issues

Individual variability is a critical factor influencing the safety and efficacy of gene therapy for hereditary hematological disorders, such as SCD, β-thalassemia, Fanconi anemia, and inherited bone marrow failure syndromes. This variability may arise from multiple sources, including genetic background, immune status, age, and disease severity. Patients with different hematological disorders harbor diverse mutation types. For example, more than 350 distinct mutations have been reported in β-thalassemia.^104^ These differences in genetic background directly affect the design efficiency and specificity of gene editing tools. Furthermore, genomic sequence variations among individuals may introduce sequences homologous to the target sites of gene editing, thereby increasing the risk of off-target editing. This risk is especially critical for the treatment of hematological disorders that rely on precise correction of HSC function.^125^^,^^126^

Beyond these technical challenges, the clinical implementation of gene therapy for hereditary hematological disorders raises distinct ethical and social controversies, particularly concerning equitable access, long-term patient monitoring, and reproductive decision-making.

Equity in access remains a primary concern. Currently approved gene therapies for SCD and β-thalassemia, such as Casgevy, are among the most expensive medical interventions worldwide, with a single treatment costing millions of dollars. This creates a substantial treatment gap between developed and developing regions. Notably, the global distribution of SCD and thalassemia is highly uneven. More than 70% of children born with severe forms of these disorders are born in developing countries, including regions in sub-Saharan Africa, India, and the Middle East.^127^ However, patients in these areas face severe barriers to accessing these potentially curative treatments and often must rely on palliative supportive care, such as blood transfusions and iron chelation. This disparity risks exacerbating global health inequities, confining life-saving innovations to wealthy nations.

Informed consent constitutes another major ethical challenge. For patients with hereditary hematological disorders, particularly children and their families, the complexity and long-term nature of gene therapy make obtaining genuinely informed consent extremely difficult. Patients and their families need to understand not only the short-term toxicities associated with myeloablative conditioning, including infertility risks and the potential for secondary malignancies, but also the long-term uncertainties that may arise from gene editing or vector integration, such as clonal hematopoiesis and leukemic transformation.^128^ These risks have been sporadically reported in some clinical trials of lentiviral vector-based therapies for blood disorders. Moreover, for young patients with reproductive plans, informed disclosure of the treatment’s impact on their germ cells and the heritable risks to offspring is particularly critical.

Given the typically long-term or even lifelong effects of gene therapy, long-term follow-up for safety evaluation is a core ethical requirement for clinical translation. Current long-term data are derived mainly from early clinical trials, and the long-term safety of newly approved therapies still requires systematic validation. For hereditary hematological disorders such as SCD and β-thalassemia, long-term follow-up to detect late-onset adverse events is generally considered to require a combination of peripheral blood chimerism monitoring, clonal lineage tracking, and annual bone marrow evaluation, each addressing distinct aspects of safety assessment.^129^ However, such intensive monitoring poses substantial logistical and financial challenges. Many patients from developing countries lack access to specialized centers for long-term assessment, and loss to follow-up may obscure delayed genotoxicity signals. Therefore, establishing a standardized and globally accessible long-term follow-up framework is an urgent ethical priority, requiring clear definitions of monitoring requirements, allocation of financial responsibilities, and strategies to ensure sustained patient participation.

Reproductive ethics and intergenerational justice represent unavoidable topics in the context of gene therapy for hereditary hematological disorders. Because many of these disorders, such as thalassemia and SCD, follow Mendelian inheritance patterns, patients and their family members often face complex reproductive decisions. Although gene therapy can cure the disease phenotype in the treated individual, it does not correct the pathogenic mutations in the patient’s germline. This means that the patient’s offspring may still inherit the genetic disorder. Therefore, clinicians have a responsibility to clearly inform patients of this limitation before treatment and to discuss assisted reproductive options, including prenatal diagnosis, preimplantation genetic testing (PGT), or germline gene editing (the latter being currently subject to international moratoria). This discussion requires a balance between respect for patient autonomy and the avoidance of exacerbating genetic discrimination.

### Future trends and prospects of rare disease therapy

#### Synergistic application of epigenetic regulation and gene editing technology

The synergy between epigenetic regulation and gene editing technologies enables bidirectional empowerment of precise DNA sequence editing and dynamic gene expression regulation, overcoming the limitations of individual technologies. Traditional gene editing focuses on permanent modification of the DNA sequence of target genes, while epigenetic editing tools (e.g., dCas9-TET1 and EpiReg-T) realize switch-like control of gene expression through reversible regulation of DNA methylation and histone modifications. Of note, catalytically inactive base editor variants (e.g., dABE8e, which utilizes a dCas9-based DNA-binding platform) can also serve as foundational tools for such epigenetic modulation when fused with transcriptional effectors, enabling reversible gene regulation without introducing DNA DSBs. The combination of the two further improves the safety and efficacy of treatment.

At present, this synergistic strategy has shown great potential in the treatment of β-hemoglobinopathies, but its clinical translation still faces three core bottlenecks. First, the long-term durability of epigenetic modifications is insufficient, and there is a risk of recurrence of the methylation status of the target gene after treatment, which may lead to a decrease in HbF expression and compromise long-term efficacy. Second, the transfection efficiency of the complex delivery system is inadequate. Existing viral or non-viral vectors still have room for optimization in terms of transfection efficiency targeting HSCs, *in vivo* retention time, and biosafety, which limits the widespread application of the therapy. Third, it is more difficult to control the off-target risk of multi-target synergistic intervention, and the combined action of CRISPR-Cas9 and dCas9-TET1 may induce abnormalities in gene sequences or epigenetic modifications in non-target regions, with potential carcinogenic risks.^81^

Over the next 5–10 years, as these technical bottlenecks are progressively resolved, the first combination therapy integrating gene editing and epigenetic regulation for β-hemoglobinopathies is expected to gain clinical approval, marking a new milestone in the cure of such hereditary hematological diseases.

#### AI in the treatment of rare diseases

AI is playing an increasingly critical role in the R&D and clinical translation of gene therapy for rare hereditary hematological disorders, and has become an indispensable tool for addressing key technical bottlenecks in this field. In drug development, AI’s predictive capabilities can substantially reduce the resources consumed in the research and development process. In 2024, the novel AI-driven protein structure prediction model AlphaFold3 was launched. Compared with previous models, AlphaFold3 has improved predictive accuracy by 50% or more, enabling the exploration of previously unknown therapeutic targets.^130^ Personalized therapy represents the ultimate goal of AI in rare disease treatment. Given the high genetic heterogeneity of rare diseases, where the same disease may be caused by different gene mutations, this therapeutic approach is particularly crucial. By analyzing patients’ genetic information and integrating clinical phenotypes, individualized treatment plans can be tailored for patients.

#### Large-scale production and industrialization of gene therapy vectors

Large-scale, low-cost vector production is the core bottleneck for the industrialization of gene therapy for hereditary hematological disorders. LV and AAV are plagued by low yield, complex preparation and stringent quality control, while engineered AAV serotypes (e.g., BBM-H901 for Xinjiuning) lack standardized mass production processes. Suspension culture and AI-assisted purification have optimized viral vector production efficiency, and LNPs as non-viral vectors boast unique industrial advantages with simple chemical synthesis and low costs, as exemplified by LNP168’s *in vivo* editing application. A unified quality control system and industry-university-research integration are critical guarantees. For China’s domestic research in particular, combining self-developed vector technology with Good Manufacturing Practice (GMP) production capacity accelerates localized industrialization and cost reduction.

#### Strategies to improve the accessibility of gene therapy

Ultra-high treatment costs, uneven medical resource distribution and technical barriers severely limit the clinical accessibility of gene therapy for hereditary hematological disorders, with international products priced far beyond the affordability of most patients. Technological optimization is the fundamental solution: vector industrialization and universal gene therapy strategies cut production and clinical costs effectively.^131^ Policy support is a key guarantee—including products in rare disease medical insurance, implementing effect-based payment and orphan drug incentives, and for developing countries, independent R&D (e.g., China’s Xinjiuning) breaks patent barriers and reduces pricing. Rational resource allocation, such as constructing regional gene therapy centers and integrating genetic detection with therapy, further improves accessibility in regions with uneven development. Notably, accessibility improvement must be based on guaranteed safety and efficacy; with technological progress, policy optimization and global resource integration, gene therapy will gradually shift from personalized high-end treatment to large-scale clinical popularization.

## Acknowledgments

This work is supported by 10.13039/501100012166National Key Research and Development Program of China (2021YFC1005303), 10.13039/501100001809National Natural Science Foundation of China (82101954, 82302101), 10.13039/501100002858China Postdoctoral Science Foundation (2023M740023), 10.13039/501100003995Anhui Provincial Natural Science Foundation (2308085QH244), Postdoctoral Researchers Foundation of Anhui Province (2022A574), University Natural Foundation of Anhui Educational Committee (2022AH051161), and Research Fund of Anhui Institute of Translational Medicine (2023zhyx-C23). The graphical abstract in this manuscript was created using BioRender.com.

## Author contributions

Z.C. contributed to conceptualization, writing – original draft and visualization; W.W. contributed to conceptualization, writing – original draft and investigation; Y.W. contributed to writing – original draft and data curation; Y.Q. contributed to investigation, validation and visualization; W.C. contributed to conceptualization, supervision, writing – review and editing and funding acquisition; F.L. contributed to conceptualization, supervision, writing – review and editing, project administration and funding acquisition. All authors have read and approved the final version of the manuscript.

## Declaration of interests

The authors declare no competing interests.

## Declaration of generative AI and AI-assisted technologies in the writing process

During the preparation of this work, the authors used DeepSeek in order to assist with English-language polishing and readability improvement. After using these tools, the authors reviewed and fully edited all content as needed and take full responsibility for the content of the publication.

## Supplemental information

Document S1. Table S1

Document S2. Article plus supplemental information
