# Supplementary material for: Gene therapy for hereditary hematological disorders: From clinical breakthroughs to future horizons
Source: Mol Ther Nucleic Acids. 2026 Aug 27;37(4):103075. doi: 10.1016/j.omtn.2026.103075 (PMC13586514; doi:10.1016/j.omtn.2026.103075)
Supplement: Supplementary file 1 [file mmc1.pdf]

OMTN, Volume 37

Supplemental information

Gene therapy for hereditary hematological disorders: From clinical breakthroughs to future horizons

Zhenghe Chen, Wei Wang, Youlan Wu, Yawen Qiang, Weisheng Cheng, and Fang Liu

Table S1 *Ex Vivo* Clinical Trials for Hemophilia

NCT Number	Other Study ID Numbers	Status	Enrollment	Interventions	Sponsor	Study Start
NCT05265767	CSCR-CMC	Phase 1|Completed	6	Auto $CD34^+$ PBSC transduced with an LV encoding a novel coagulation factor VIII transgene	Christian Medical College, Vellore, India	2022/4/1
NCT04418414	ET3-201	Phase 1|Not yet recruiting	7（Estimated）	Transplantation of autologous $CD34^+$ hematopoietic stem cells transduced *ex vivo* with the CD68-ET3 lentiviral vector.	Expression Therapeutics	2024/9/1
NCT03818763	PRO00033763	Phase 1|Recruiting	5	Auto $CD34^+$ PBSC, transduced with an LV encoding the B domain deleted from human coagulation factor VIII	Medical College of Wisconsin	2020/4/29
